# Modelling innate and adaptive immune responses in whole blood: A modified *ex vivo* assay without anticoagulants and synthetic media

**DOI:** 10.14440/jbm.0122

**Published:** 2025-09-10

**Authors:** Victor I. Seledtsov, Tatyana Y. Lyubavskaya, Anatoly A. Pyshenko, Alexei von Delwig, Irina A. Seledtsova

**Affiliations:** Russian Scientific Centre for Surgery named after Academician B. V. Petrovsky, Federal State Budgetary Scientific Institution, Moscow 119991, Russia

**Keywords:** Whole blood immunoreactivity assay, Innate immunity, Adaptive immunity, Cytokines, Immune status

## Abstract

**Background:**

Blood is central to immune defense, rendering accurate assessment of its immunoreactivity vital for medical and biotechnological applications.

**Objective:**

This study presented a novel whole blood immunoreactivity assay (WBIA) designed to mimic natural physiological conditions, preserving essential cell–cell and cell–cytokine interactions for *ex vivo* immunological analysis.

**Methods:**

Fresh whole blood (with or without heparin) was stimulated with lipopolysaccharide (LPS), concanavalin A (Con A), or both, activating innate and adaptive immunity. Cytokine levels were measured through enzyme-linked immunosorbent assay after incubation.

**Results:**

Coagulation enhanced secretion of interleukin (IL)-2 and vascular endothelial growth factor (VEGF) in mitogen-stimulated samples. LPS induced tumor necrosis factor (TNF)-α, IL-6, and VEGF, while LPS + Con A co-stimulation produced the highest levels of interferon (IFN)-γ, IL-2, and IL-10. Peak cytokine concentrations were reached at 18 h, declining by 48–72 h. In 18 h LPS + Con A-stimulated serum blood samples from 30 healthy donors (19 women, 11 men, aged 30–55), cytokine levels (pg/mL, mean ± standard error of the mean) were as follows: IL-1β at (521 ± 62), IL-2 (24 ± 4), IL-6 (569 ± 43), IL-8 (277 ± 28), IL-10 (198 ± 35), IL-18 (293 ± 19), IFN-γ (227 ± 108), TNF-α (930 ± 126), and VEGF (655 ± 55).

**Conclusion:**

The WBIA provides a reliable, physiologically relevant model for evaluating immune responses to stimuli. Its high fidelity to *in vivo* conditions makes it a valuable tool for testing immunomodulatory drugs and monitoring immune status in clinical settings.

## 1. Introduction

The immune system orchestrates a highly complex set of responses to detect, contain, and eliminate pathogens, relying on tightly regulated coordination between innate and adaptive mechanisms. A comprehensive understanding of these immunological processes is crucial for improving the precision of diagnostics, vaccine development, immunotherapy, and personalized medicine.

Circulating blood serves as a critical conduit for immune surveillance, facilitating the trafficking of leukocytes and the distribution of cytokines, chemokines, and soluble pattern recognition molecules. However, the immunobiological role of blood extends beyond transport—it functions as a dynamic immunological tissue capable of mounting active responses to systemic stimuli. Consequently, the ability to assess immune reactivity in whole blood, under conditions that preserve physiological integrity, is increasingly recognized as a valuable tool in both research and clinical settings.

Whole blood immunoreactivity assays (WBIAs) provide an *ex vivo* platform to probe immune functionality in a more integrative and systemic context compared to isolated cell models. Unlike purified peripheral blood mononuclear cell (PBMC) assays, WBIA retains the native liquid and cell environment, including red blood cells, platelets, and coagulation pathways, all of which are involved in the regulation of immune responses.^1^ Notably, alterations in blood immune reactivity have been implicated in disease progression across a spectrum of conditions—from infectious diseases and autoimmune disorders to cancer and trauma-related immunosuppression. The impact of these processes must be considered when objectively analyzing the immunoreactivity of blood, which serves as a critical barrier against pathogenic invasion.^2^

Historically, WBIA protocols have relied on anticoagulants (*e*.*g*., heparin, ethylenediaminetetraacetic acid, and citrate) and synthetic culture media to preserve sample stability during incubation.^1^-^7^ While effective for logistical purposes, these components introduce significant deviations from physiological norms. Heparin, in particular, exhibits diverse biological activities that go far beyond its anticoagulant function. It interacts with a wide array of immune mediators and has been shown to influence several processes, including angiogenesis, leukocyte trafficking, and cytokine binding.^8^,^9^ These pleiotropic effects may confound the interpretation of functional immune readouts and compromise the validity of WBIA results.

In contrast, enhanced blood coagulation is typically associated with pathogen-induced disturbances in the microcirculatory bed. The primary physiological role of coagulation is to maintain tissue perfusion and vascular integrity. In addition, it facilitates interactions between platelets and leukocytes—pivotal processes in immune surveillance and the containment of pathogen spread.^10^ Recent studies have demonstrated that coagulation-derived mediators can regulate inflammation by exerting direct effects on immune cell activation,^11^,^12^ further reinforcing the rationale for minimizing anticoagulant use in assays intended to model true immune physiology.

In parallel, synthetic culture media often contain non-physiological concentrations of glucose, amino acids, and buffering agents, which can alter the metabolism and functional phenotypes of immune cells. Accordingly, WBIA models that omit both anticoagulants and synthetic additives are more likely to replicate *in vivo* immune dynamics and provide biologically accurate insights into immune function.

In this study, we reported a modified WBIA protocol designed to more accurately represent the physiological conditions of circulating blood. The WBIA protocol we propose omits both heparin, which interferes with contact-dependent immune interactions, and synthetic culture additives, which may artificially modify cellular metabolism and cytokine secretion.

Previous applications of WBIA have predominantly focused on assessing antigen-specific immune reactivity,^13^-^17^ which may not fully capture the broader spectrum of immunobiological responses required for protection against diverse pathogens. In our study, innate and adaptive immune responses were stimulated in whole blood samples from healthy donors using lipopolysaccharide (LPS), a toll-like receptor 4 (TLR4) ligand, and concanavalin A (Con A), a well-established T-cell mitogen.

Our objective was to characterize the cytokine secretion profiles elicited by this dual stimulation—focusing on tumor necrosis factor-alpha (TNF-α), interleukin (IL)-6, IL-2, IL-10, interferon-gamma (IFN-γ), and vascular endothelial growth factor (VEGF)—within a whole blood system that preserves the structural and functional complexity of the *in vivo* milieu.

This approach aligns with a growing body of evidence underscoring the importance of maintaining the native hemostatic and biochemical environment when modeling immune responses *ex vivo*.^18^ It also supports the broader goal of developing functionally relevant, standardizable immunoreactivity assays applicable to translational research, immunotoxicity screening, and personalized immune profiling.

## 2. Materials and methods

The study was approved by the local ethics committee of the Petrovsky National Research Centre of Surgery (protocol ID: 9, October 2024). All blood donors were informed about the use of their blood for research purposes, and written informed consent was obtained.

### 2.1. Whole blood immunoreactivity assay

Venous blood was collected from healthy donors aged 30–55 years into pre-cooled vacuum tubes, either containing heparin or having no anticoagulant. Immediately after collection, the blood tubes were placed on ice. Subsequently, the blood from each tube was divided into four equal aliquots (2 mL each). To one aliquot, 50 μL of physiological saline was added; to the second, 50 μL of LPS from *Salmonella* Typhi (Pyrogenal, Medgamal, Russia; final concentration 1 μg/mL); to the third, 50 μL of Con A (Sigma-Aldrich, USA; final concentration 10 μg/mL); and to the fourth, a combination of LPS and Con A. All samples were incubated at 37.5°C for the designated periods. To obtain plasma or serum, the tubes were centrifuged at 2,000 g for 20 min, after which the supernatants (cell-free fluids) were aliquoted (0.5 mL/icrotube) and stored frozen until further analysis.

### 2.2. Determination of cytokine levels in plasma and serum

The concentrations of IL-1β, IL-2, IL-6, IL-8, IL-10, IL-18, TNF-α, IFN-γ, and VEGF in the samples were determined using enzyme-linked immunosorbent assay (ELISA) (Vector-Best, Russia), according to the manufacturer’s instructions.

### 2.3. Statistical analysis

The data are presented as mean ± standard error of the mean. The significance of differences between samples obtained from the same donor cohort was assessed using the paired Student’s *t*-test. Differences were considered statistically significant at *p*<0.05.

## 3. Results

### 3.1. Effects of LPS and Con A on the secretion of cytokines by blood cells in serum and plasma samples

In this study, two activators were used to stimulate blood cells: LPS and Con A. LPS is a thermostable component of the outer membrane of all gram-negative microorganisms. It binds to the cluster of differentiation 14/toll-like receptor 4/myeloid differentiation protein 2 receptor complex, primarily located on phagocytic cells and B lymphocytes, and acts as a potent activator of innate immunity.^19^ Con A is a plant lectin capable of binding to structures containing α-D-mannosyl and α-D-glucosyl residues. It activates lymphocytes, predominantly T-cells, and thus serves as an antigen-nonspecific activator of adaptive immunity.^20^

**Figure 1:**
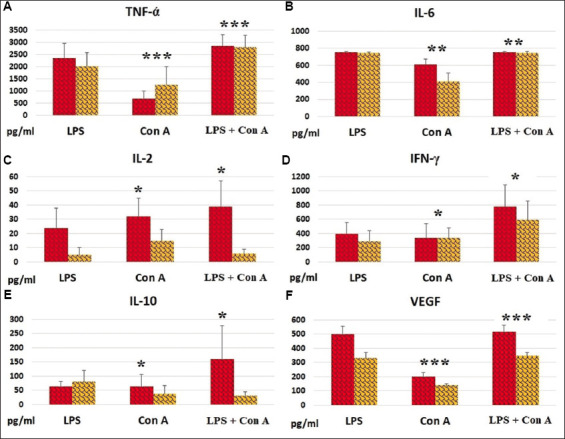
Cytokine levels (pg/mL) in mitogen-stimulated blood samples. Blood was stimulated with the indicated mitogens for 18 h. The content (mean ± standard error of the mean) of cytokines in serum samples without heparin (red bars) and plasma samples with heparin (yellow bars) was measured. (A) TNF-α (number of donor samples *n* = 7); (B) IL-6 (*n* = 7); (C) IL-2 (*n* = 5); (D) IFN-γ (*n* = 5); (E) IL-10 (*n* = 7); (F) VEGF (*n* = 7) Notes: *p<0.05; **p<0.03; ***p<0.01. Abbreviations: Con A: Concanavalin A; IFN: Interferon; IL: Interleukin; LPS: Lipopolysaccharide; TNF-α: Tumor necrosis factor-alpha; VEGF: Vascular endothelial growth factor.

Figure 1A-F shows the effects of the mitogens on cytokine secretion by blood cells in the absence or presence of the anticoagulant heparin. The concentration of TNF-α in unstimulated blood samples did not exceed 1000 pg/mL. LPS induced a significant increase in the cellular secretion of this cytokine (*p*<0.01), whereas Con A had no substantial effect. Similar findings were observed in both serum samples (without heparin) and plasma samples (with heparin) (Figure 1A).

The concentration of IL-6 in unstimulated blood samples was no more than 400 pg/mL. As with TNF-α, LPS, but not Con A, significantly enhanced IL-6 secretion by blood cells (*p*<0.05). The combined effect of LPS and Con A was not greater than the effect induced by LPS alone (Figure 1B).

The concentration of IL-2 in unstimulated blood samples was low (<2 pg/mL). Both LPS and Con A markedly increased IL-2 secretion. Interestingly, the greatest increase in IL-2 levels was observed in serum samples stimulated simultaneously with LPS and Con A (*p*<0.05 compared to plasma samples) (Figure 1C).

Similar to IL-2, the concentration of IFN-γ in unstimulated blood samples was low (<2 pg/mL). Both activators, LPS and Con A, significantly elevated the secretion of IFN-γ. The greatest increase in IFN-γ secretion was observed following simultaneous stimulation with LPS and Con A, indicating an additive effect of the two activators (Figure 1D).

The concentration of IL-10 in unstimulated blood samples was below 20 pg/mL. LPS and Con A each moderately stimulated IL-10 secretion by blood cells to a comparable extent. The most pronounced increase in IL-10 secretion was detected in serum samples stimulated with both LPS and Con A. In contrast, IL-10 levels in plasma samples remained relatively low **(**Figure 1E).

The concentration of VEGF in unstimulated blood samples did not go beyond 100 pg/mL. In both plasma and serum samples, LPS more effectively stimulated VEGF secretion. VEGF levels were notably higher in serum samples than in their plasma counterparts (Figure 1F).

### 3.2. Time-dependent changes in cytokine levels in serum samples from mitogen-stimulated blood cells

**Figure 2:**
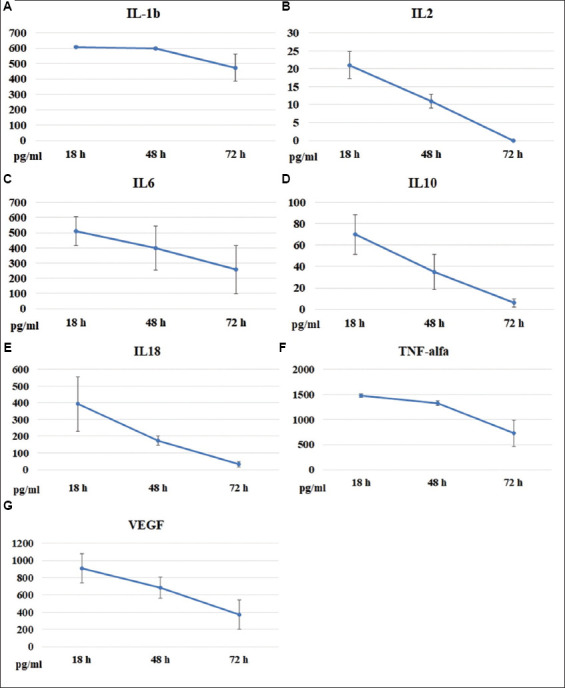
Time-dependent changes in cytokine levels (pg/mL) in serum samples. Blood samples obtained from donors (*n* = 6) were stimulated with mitogens (LPS + Con A). The cytokine contents (mean ± SEM) were measured at the indicated times after incubation with the mitogens. (A) IL-1β; (B) IL-2; (C) IL-6; (D) IL-10; (E) IL-18; (F) TNF-α; (G) VEGF. Abbreviations: Con A: Concanavalin A; IL: Interleukin; LPS: Lipopolysaccharide; TNF-α: Tumor necrosis factor-alpha; VEGF: Vascular endothelial growth factor.

Blood samples obtained from 5 donors (*n* = 5) were incubated with LPS + Con A. The levels of IL-1β, IL-2, IL-6, IL-10, IL-18, TNF-α, and VEGF in serum samples were determined at 18, 48, and 72 h after the onset of incubation. According to the data presented in Figure 2A-G, the highest cytokine levels in serum were observed in the 18-h samples. Subsequently, at 48 and 72 h, cytokine concentrations in all samples showed a clear decreasing trend.

Table 1 lists the numerical data on cytokine levels in serum samples obtained from 30 healthy donors (19 women and 11 men). All blood samples were stimulated with LPS + Con A for 18 h.

**Table 1:** Cytokine levels (Mean ± SEM) in serum samples (*n* = 30) simulated with LPS and Con A

Cytokine	Concentrations (pg/mL), Mean ± SEM, minimum–maximum
IL-1β	521±62, 10–979
IL-2	24±4, 5–85
IL-6	569±43, 10–774
IL-8	277±28, 10–965
IL-10	198±35, 10–1070
IL-18	293±19, 175–588
IFN-γ	227±108, 10–1492
TNF-α	930±126, 10–3486
VEGF	655±55, 153–1634

Abbreviations: Con A: Concanavalin A; IFN: Interferon; IL: Interleukin; LPS: Lipopolysaccharide; M ± SEM: Mean ± standard error of the mean; TNF-α: Tumor necrosis factor-alpha; VEGF: Vascular endothelial growth factor.

## 4. Discussion

Blood immunoreactivity plays a decisive role in shaping the course of inflammatory responses in the body. Under physiological conditions, these mechanisms protect against pathogens; however, when dysregulated, they contribute to the development of pathological states. Therefore, a physiologically relevant *ex vivo* model to assess blood immune reactivity is of great importance for both research and clinical applications.

Previous applications of the WBIA have predominantly focused on assessing antigen-specific immune responses^13^-^17^ and cytokine production,^21^ particularly in the contexts of infectious diseases and in terms of vaccine efficacy. For instance, WBIA has been employed to evaluate dual anti-CD28 and anti-CD49d co-stimulation for comprehensive analysis of both antigen-specific T-cell functions and complex intercellular interactions in response to various fungal and viral antigens.^22^ Similarly, WBIA has been used to assess immune responses to influenza and pertussis vaccines, providing insights into cytokine release profiles and correlates of protection following immunization.^14^,^15^ During the COVID-19 pandemic, WBIA played a critical role in evaluating SARS-CoV-2-specific T-cell responses in both vaccinated individuals and cancer patients, underscoring its utility in immunocompromised populations.^15^ Beyond infectious diseases, WBIA has also been utilized for identifying immunotoxicity hazards.^23^

WBIA offers several advantages over traditional PBMC-based assays, including reduced processing time and cost. Conventional PBMC assays remove plasma, granulocytes, platelets, and clotting factors, thereby disrupting critical immunomodulatory interactions. In contrast, WBIA preserves the integrative nature of blood physiology, which includes thrombocyte-derived chemokines and complement-mediated priming.

However, virtually all previous studies using WBIA have relied on heparinized blood and often used synthetic media, limiting its physiological relevance. Heparin is a negatively charged, linear, heterogeneous glycosaminoglycan of relatively low molecular weight, composed of various disaccharide-repeating units connected through α-1,4 glycosidic bonds and containing carboxylate, acetamido, sulfamido, and sulphate groups, as well as occasional free amines.^24^ Heparin’s ability to bind various cytokines and chemokines—driven by electrostatic interactions between its negatively-charged sulphate groups and positively-charged amino acids on proteins—can impact their biological activity by altering half-life, availability, and receptor affinity. These non-anticoagulant effects can artificially dampen immune responses in *ex vivo* assays.^25^ Heparin can also inhibit the activity of key cytokines and chemokines involved in inflammation, including chemokine (C-X-C motif) ligand 1, IL-6, and IL-8—important factors in acute respiratory distress syndrome—thereby potentially distorting the inflammatory response.^26^ Moreover, heparin suppresses the secretion of critical pro-inflammatory cytokines, such as IL-2 and IFN-γ, and inhibits inflammatory responses coupled with phagocytosis.^27^ It also affects the maturation and antigen-presenting functions of dendritic cells, influencing their ability to initiate and regulate immune responses.^28^ Specifically, heparin can enhance IL-12 activity, potentially through stabilization of the IL-12 heterodimer and improved receptor binding, while modulating production of TNF-α and other cytokines.^29^ Notably, at concentrations required to prevent clotting *in vitro*, heparin disrupts intercellular adhesion by altering membrane charges and binding surface molecules, impairing immunological synapse formation and T-cell activation.^9^ Given these findings, WBIA protocols aiming to replicate physiological immune dynamics should minimize or eliminate the use of heparin to avoid such confounding effects and preserve the native immunoreactivity of whole blood.

It is also evident that synthetic media—containing high, non-physiological concentrations of vitamins and growth factors—may exert artificial effects on immune cell function and phenotype.^30^ Standard media, such as RPMI-1640 or DMEM, include supraphysiological concentrations of glucose, amino acids, vitamins, and buffering agents, which can alter metabolism, differentiation, and activation profiles of immune cells.^31^ For example, high glucose levels have been shown to skew macrophage polarization and dampen production of pro-inflammatory cytokines, such as TNF-α and IL-6.^32^,^33^ Consequently, synthetic media may lead to misleading conclusions when used to assess immunoreactivity, particularly in assays aimed at predicting clinical outcomes or therapeutic responses.

We believe that our WBIA, using coagulating blood without synthetic additives, offers more physiologically relevant conditions than assays employing heparin and synthetic media, which interfere with intercellular immunological interactions and may unnaturally affect immune cell functionality. Indeed, in the experiments presented here, heparin markedly reduced the secretion of IL-2 and VEGF by blood cells.

Unlike conventional assays, our model excludes synthetic culture additives and anticoagulants to preserve native cell–cell interactions. Where necessary, heparin was used solely to study the role of clotting in immune responses. Blood samples were exposed to two classical mitogens—LPS and Con A—to simultaneously stimulate innate and adaptive immune pathways. LPS, a major component of the outer membrane of Gram-negative bacteria, engages innate immune cells by first binding LPS-binding protein in plasma, then forming a complex with CD14 and interacting with TLR4 and MD-2. This cascade triggers robust pro-inflammatory cytokine production, including TNF-α, IL-6, and IL-1β.^19^ Meanwhile, Con A, a plant lectin from *Canavalia ensiformis*, activates T cells by cross-linking surface glycoproteins, promoting proliferation and cytokine secretion. Con A remains extensively used as a tool to assess T-cell function and activation.^20^

A complex cytokine network mediates the dynamic interplay between innate and adaptive immunity. Pro-inflammatory mediators (*e.g*., TNF-α, IL-1β, IL-6, IL-8, IL-18) and anti-inflammatory or regenerative cytokines (*e.g*., IL-10, VEGF) regulate the magnitude and duration of immune responses. Perturbations in this balance are associated with immunopathological conditions.^34^,^35^ Of particular interest is IL-2, which exhibits both pro- and anti-inflammatory effects and is critical for maintenance of immune memory.^36^,^37^

Our results align with the known biology of these mitogens: LPS predominantly stimulated production of cytokines associated with innate immune activation (TNF-α, IL-6, IL-10, VEGF), while combined stimulation with LPS and Con A elicited the highest levels of adaptive cytokines, such as IL-2 and IFN-γ. These findings reinforce the notion that innate immune activation is a pre-requisite for effective adaptive immune responses.

Interestingly, we observed that clotting enhanced secretion of IL-2, IL-10, and VEGF, suggesting a critical role for cell–cell contacts and hemostatic factors in modulating cytokine production, and underscoring the physiological importance of clotting in immune regulation.^38^

The temporal kinetics of cytokine release showed a peak at 18 h post-stimulation, followed by a decline at later time points. This likely reflects a combination of reduced cellular secretion and protease-mediated degradation of cytokines in serum. The short optimal incubation period is advantageous, minimizing *ex vivo* artefacts, such as microbial contamination or leukocyte apoptosis.

Our data also highlight that unstimulated control samples consistently exhibited very low cytokine concentrations—often below detection limits—making graphical comparisons and stimulation index calculations impractical. This observation supports the robustness of our stimulation protocol and reinforces the physiological silence of resting blood under controlled conditions.

A skin immunological test with LPS and Con A, performed without the use of heparin or synthetic media, could, to some extent, be considered an *in vivo* analogue of the WBIA described in this study. However, the use of such skin tests with mitogens is neither certified nor legally regulated, and carries the risk of adverse effects on the human body.

The WBIA described here could be particularly valuable for: (i) Evaluating immunomodulatory effects of pharmacological agents; (ii) Monitoring immune competence in patients receiving immunotherapy; (iii) Assessing immune dysfunction in autoimmune diseases, infections, or immunodeficiencies; (iv) Predicting outcomes in critical illnesses, such as sepsis or COVID-19; and (v) Investigating cytokine signatures associated with effective vaccination responses. Moreover, this approach may prove valuable in low-resource or high-containment environments. For example, whole blood assays have been adapted for microgravity research aboard the International Space Station,^6^ where minimal handling and preservation of native conditions are crucial.

The simplicity of WBIA—requiring minimal manipulation and no synthetic culture media—enhances its translational potential in both clinical and research settings. It preserves the native milieu of blood, maintaining essential cell–cell and cell–matrix interactions, which tend to be disrupted in traditional assays. In addition to diagnostics, the WBIA platform could facilitate therapeutic modulation of blood components. Short-term pre-conditioning of blood with immunomodulators, as tested in our model, may enhance the therapeutic potential of transfused cellular or plasma products by priming their immune functions.

Nonetheless, the present study is subject to limitations. The diagnostic and prognostic value of WBIA in clinical populations remains to be established. Comparative analyses between healthy donors and patients with immunopathological disorders will be crucial. Preliminary data from our ongoing studies suggest WBIA profiles vary significantly with disease severity and therapeutic interventions, particularly in autoimmune diseases. It is also important to note that cytokine concentrations can vary depending on the ELISA kits and reagents used. Thus, establishing standardized protocols and reference ranges is critical for broader clinical adoption.

## 5. Conclusion

Our modified WBIA offers a physiologically relevant, simple, and informative approach to assess the immunobiological potential of blood. By avoiding the use of synthetic culture media and anticoagulants, the assay preserves native immune dynamics. This is particularly valuable in contexts where maintaining the integrity of the cellular microenvironment and soluble mediators is critical. By replicating *in vivo*-mimicking conditions, WBIA provides a functional and integrated snapshot of both innate and adaptive immune responses, offering clear advantages over traditional PBMC- or serum-based methods.

The method holds significant promise for studying the effects of immunomodulatory agents, monitoring immune health, and potentially predicting clinical outcomes across various diseases. Its simplicity—requiring minimal manipulation, no cell separation, and no artificial additives—renders it especially suitable for clinical diagnostics, vaccine trials, and immunity monitoring in field or resource-limited settings.

Importantly, the assay could be extended to the stratification of patients in terms of immune competence or inflammatory risk, potentially informing personalized treatment strategies. For example, WBIA may help identify individuals with attenuated cytokine responses, whether due to immunosuppressive therapy, chronic infections, or age-related immune decline.

Future applications may include integration with high-throughput multiplex cytokine platforms and machine learning classifiers to identify disease-specific immune signatures. Such innovations would further enhance the diagnostic, prognostic, and research utility of the assay across a wide range of immunity-mediated conditions.

In summary, WBIA is not only a technically accessible method but also a conceptually robust model that bridges experimental immunology and clinical relevance. Its successful adoption in translational settings will benefit from continued endeavors toward standardization and multicenter validation.

## Data Availability

No datasets were generated or analyzed during the present study.
